# 2,6-Dichloro-1-[(1*E*)-2-(phenyl­sulfon­yl)ethen­yl]benzene

**DOI:** 10.1107/S1600536811011901

**Published:** 2011-04-07

**Authors:** Michael S. South, Adirika J. Obiako, Richard E. Sykora, David C. Forbes

**Affiliations:** a Department of Chemistry, University of South Alabama, Mobile, AL 36688-0002 USA

## Abstract

In the title compound, C_14_H_10_Cl_2_O_2_S, the product of a base-catalyzed condensation followed by deca­rboxylation of the carboxyl­ate group of the sulfonyl derivative, the configuration of the alkene unit is *E*. The torsion angle between the alkene unit and the 2,6-dichloro­phenyl ring system is −40.8 (3)°. The dihedral angle between the rings is 80.39 (7)°.

## Related literature

For a review on the use of vinyl sulfones in organic chemistry, see: Simpkins (1990 ▶). For the use of phenyl­sulfonyl­acetic acid in the formation of vinyl sulfones, see: Baliah & Seshapathirao (1959 ▶). For a general review on the condensation of activated methyl­enes onto aryl aldehydes, see: Jones (1967 ▶). For the structure of the related phenyl vinyl sulfone, see: Briggs *et al.* (1998 ▶).
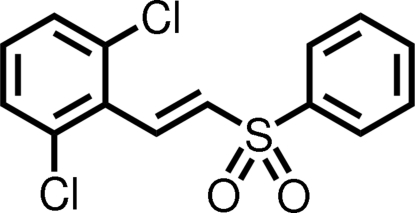


## Experimental

### 

#### Crystal data

C_14_H_10_Cl_2_O_2_S
*M* *_r_* = 313.18
Triclinic, 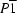

*a* = 7.5924 (6) Å
*b* = 8.3060 (4) Å
*c* = 11.3360 (9) Å
α = 78.639 (5)°
β = 84.976 (7)°
γ = 77.497 (6)°
*V* = 683.49 (8) Å^3^
*Z* = 2
Mo *K*α radiation
μ = 0.62 mm^−1^
*T* = 290 K
0.52 × 0.34 × 0.06 mm

#### Data collection

Oxford Xcalibur E diffractometer
Absorption correction: analytical (*CrysAlis PRO*; Oxford Diffraction, 2010 ▶) *T*_min_ = 0.810, *T*_max_ = 0.961
4291 measured reflections
2491 independent reflections
1741 reflections with *I* > 2σ(*I*)
*R*_int_ = 0.017

#### Refinement

*R*[*F* ^2^ > 2σ(*F* ^2^)] = 0.033
*wR*(*F* ^2^) = 0.078
*S* = 0.95
2491 reflections
173 parameters
H-atom parameters constrained
Δρ_max_ = 0.20 e Å^−3^
Δρ_min_ = −0.22 e Å^−3^

### 

Data collection: *CrysAlis PRO* (Oxford Diffraction, 2010 ▶); cell refinement: *CrysAlis PRO*; data reduction: *CrysAlis PRO*; program(s) used to solve structure: *SHELXS96* (Sheldrick, 2008 ▶); program(s) used to refine structure: *SHELXL96* (Sheldrick, 2008 ▶); molecular graphics: *OLEX2* (Dolomanov *et al.*, 2009 ▶); software used to prepare material for publication: *publCIF* (Westrip, 2010 ▶).

## Supplementary Material

Crystal structure: contains datablocks I, global. DOI: 10.1107/S1600536811011901/ng5134sup1.cif

Structure factors: contains datablocks I. DOI: 10.1107/S1600536811011901/ng5134Isup2.hkl

Additional supplementary materials:  crystallographic information; 3D view; checkCIF report

## Figures and Tables

**Fig. 1.**
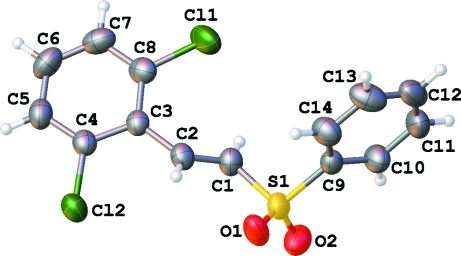
A thermal ellipsoid plot (50%) of the title compound showing the labeling scheme.

## supplementary crystallographic information

### Comment

We recently explored the use of commercially available phenylsulfonylacetic acid under base catalysis with the anticipation of observing the same mode of transfer using sulfonium salts; methylene transfer onto carbonyl derivatives. The use of not sulfonium but sulfonyl functionality does allow for one to explore catalysis, which is a realm of S-ylide chemistry yet to be fully explored. For this study, observed was not only methylene transfer but formation of the condensation adduct vinyl sulfone (an α,β-unsaturated sulfone). Under not base but acid catalysis, this type of condensation is common as previously reported by Baliah & Seshapathirao (1959) and Jones (1967). The title compound, C_14_H_10_Cl_2_O_2_S, was isolated as the major product in moderate yield and offered definitive evidence of the condensation of the 2,6-dichlorobenzaldehyde with phenylsulfonylacetic acid.

The C1–C2 bond distance of 1.320 (3) Å confirms the alkene moiety, the configuration of which is E. This distance is slightly elongated as compared with the comparable distance of 1.313 (3) Å in phenyl vinyl sulfone (PVS) reported by Briggs *et al.* (1998). Other geometric parameters in the title compound are similar but also subtly affected relative to PVS. For example, the average S=O bond lengths are 1.436 (2) Å in the title compound but 1.443 (1) Å in PVS. Also shortened are the S–C bonds in the title compound (1.7683 (19) and 1.748 (2) Å) relative to PVS (1.770 (2) and 1.755 (2) Å), the longer bond in both cases being to the phenyl moiety. The C–S–C bond is noticably more acute in the title compound (102.84 (9)°) relative to PVS (104.64 (8)°), while the O=S=O angle in PVS (118.79 (8)°) is slightly more acute than the comparable angle in the title compound (119.35 (10)°). The torsion angle between the alkene moiety and the 2,6-dichlorophenyl ring in the title compound is 40.8 (3)°.

### Experimental

To a 0.125*M* THF solution of phenylsulfonylacetic acid (1 g, 4.99 mmol, 2.0 equiv) was added 439 mg of 2,6-dichlorobenzaldehyde (2.51 mmol, 1.0 equiv). A 40 wt% solution of benzyltrimethylammonium hydroxide in methanol was next added by syringe (2.1 ml, 4.99 mmol, 2.0 equiv). The 50 ml one-neck round bottomed flask equipped with a magnetic stir bar was fitted with a condenser and allowed to warm to reflux. After a period of 18 h, the solution was cooled to 60 °C and 15 ml of deionized water was added and allowed to stir at this temperature for a period of 1 h. The resulting mixture was allowed to cool to room temperature at which time the mixture was washed with approximately 20 ml of ethyl acetate. After partitioning the organic from the aqueous phase, the organic fraction was washed with brine, dried over anhydrous magnesium sulfate, and concentrated *in vacuo*. Purification by column chromatography over silica gel (eluting with 9:1 hexanes/ethyl acetate) afforded the title compound (355 mg, 45% yield). White crystalline solid, mp: 78–82 °C. IR (KBr): 1628, 1446, 1307, 1147 cm^-1. 1^H NMR (300 MHz; CDCl_3_) δ 7.19 (2*H*, m), 7.35 (2*H*, s), 7.64 (2*H*, m), 7.84 (1*H*, d, J = 15.9 Hz), 7.98 (1*H*, brs); ^13^C NMR (300 MHz; CDCl_3_) δ 128.4, 129.5, 129.9, 131.3, 133.9, 135.3, 135.9, 136.3, 140.1; EI—MS (m/*z*) 313 (*M*+); HRMS calcd for C_14_H_10_Cl_2_O_2_S (*M*+H) 312.9857, found 312.9858.

### Refinement

Hydrogen atoms were placed in calculated positions and allowed to ride during subsequent refinement, with *U*_iso_(H) = 1.2*U*_eq_(C) and C—H distances of 0.93 Å.

### Crystal data

**Table:**

C_14_H_10_Cl_2_O_2_S	*Z* = 2
*M**_r_* = 313.18	*F*(000) = 320
Triclinic, *P*1	*D*_x_ = 1.522 Mg m^−^^3^
Hall symbol: -P 1	Mo *K*α radiation, λ = 0.71073 Å
*a* = 7.5924 (6) Å	Cell parameters from 2060 reflections
*b* = 8.3060 (4) Å	θ = 3.2–25.3°
*c* = 11.3360 (9) Å	µ = 0.62 mm^−^^1^
α = 78.639 (5)°	*T* = 290 K
β = 84.976 (7)°	Plate, colorless
γ = 77.497 (6)°	0.52 × 0.34 × 0.06 mm
*V* = 683.49 (8) Å^3^	

### Data collection

**Table:**

Oxford Xcalibur E diffractometer	2491 independent reflections
Radiation source: fine-focus sealed tube	1741 reflections with *I* > 2σ(*I*)
graphite	*R*_int_ = 0.017
Detector resolution: 16.0514 pixels mm^-1^	θ_max_ = 25.4°, θ_min_ = 3.2°
ω scans	*h* = −9→9
Absorption correction: analytical (*CrysAlis PRO*; Oxford Diffraction, 2010)	*k* = −6→10
*T*_min_ = 0.810, *T*_max_ = 0.961	*l* = −13→13
4291 measured reflections	

### Refinement

**Table:**

Refinement on *F*^2^	Secondary atom site location: difference Fourier map
Least-squares matrix: full	Hydrogen site location: inferred from neighbouring sites
*R*[*F*^2^ > 2σ(*F*^2^)] = 0.033	H-atom parameters constrained
*wR*(*F*^2^) = 0.078	*w* = 1/[σ^2^(*F*_o_^2^) + (0.0394*P*)^2^] where *P* = (*F*_o_^2^ + 2*F*_c_^2^)/3
*S* = 0.95	(Δ/σ)_max_ < 0.001
2491 reflections	Δρ_max_ = 0.20 e Å^−^^3^
173 parameters	Δρ_min_ = −0.22 e Å^−^^3^
0 restraints	Extinction correction: *SHELXL*, Fc^*^=kFc[1+0.001xFc^2^λ^3^/sin(2θ)]^-1/4^
Primary atom site location: structure-invariant direct methods	Extinction coefficient: 0.025 (2)

### Special details

**Table:**

Geometry. All e.s.d.'s (except the e.s.d. in the dihedral angle between two l.s. planes) are estimated using the full covariance matrix. The cell e.s.d.'s are taken into account individually in the estimation of e.s.d.'s in distances, angles and torsion angles; correlations between e.s.d.'s in cell parameters are only used when they are defined by crystal symmetry. An approximate (isotropic) treatment of cell e.s.d.'s is used for estimating e.s.d.'s involving l.s. planes.
Refinement. Refinement of *F*^2^ against ALL reflections. The weighted *R*-factor *wR* and goodness of fit *S* are based on *F*^2^, conventional *R*-factors *R* are based on *F*, with *F* set to zero for negative *F*^2^. The threshold expression of *F*^2^ > 2σ(*F*^2^) is used only for calculating *R*-factors(gt) *etc*. and is not relevant to the choice of reflections for refinement. *R*-factors based on *F*^2^ are statistically about twice as large as those based on *F*, and *R*- factors based on ALL data will be even larger.

### Fractional atomic coordinates and isotropic or equivalent isotropic displacement parameters (Å^2^)

**Table:**

	*x*	*y*	*z*	*U*_iso_*/*U*_eq_	
S1	0.09983 (8)	0.38328 (6)	0.16893 (5)	0.04924 (19)	
Cl1	0.28536 (9)	0.37676 (7)	0.55021 (6)	0.0761 (2)	
Cl2	0.22076 (8)	−0.20158 (6)	0.41978 (5)	0.0586 (2)	
O1	0.2065 (2)	0.25879 (17)	0.10588 (14)	0.0635 (5)	
O2	−0.0899 (2)	0.43654 (18)	0.14956 (15)	0.0668 (5)	
C1	0.1250 (3)	0.3114 (2)	0.32340 (19)	0.0453 (5)	
H1	0.0678	0.3793	0.3774	0.054*	
C2	0.2236 (3)	0.1626 (2)	0.36487 (19)	0.0429 (5)	
H2	0.2819	0.1011	0.3073	0.051*	
C3	0.2521 (2)	0.0828 (2)	0.49086 (18)	0.0386 (5)	
C4	0.2567 (3)	−0.0897 (2)	0.52656 (19)	0.0418 (5)	
C5	0.2828 (3)	−0.1734 (3)	0.6421 (2)	0.0534 (6)	
H5	0.2842	−0.2878	0.6616	0.064*	
C6	0.3067 (3)	−0.0875 (3)	0.7290 (2)	0.0628 (7)	
H6	0.3230	−0.1430	0.8082	0.075*	
C7	0.3066 (3)	0.0818 (3)	0.6987 (2)	0.0601 (7)	
H7	0.3249	0.1400	0.7572	0.072*	
C8	0.2794 (3)	0.1645 (2)	0.5823 (2)	0.0489 (6)	
C9	0.1980 (3)	0.5626 (2)	0.14040 (18)	0.0420 (5)	
C10	0.0892 (3)	0.7191 (2)	0.1258 (2)	0.0552 (6)	
H10	−0.0358	0.7315	0.1295	0.066*	
C11	0.1671 (4)	0.8583 (3)	0.1055 (2)	0.0666 (7)	
H11	0.0944	0.9651	0.0967	0.080*	
C12	0.3499 (4)	0.8396 (3)	0.0984 (2)	0.0646 (7)	
H12	0.4015	0.9338	0.0847	0.077*	
C13	0.4585 (3)	0.6833 (3)	0.1112 (2)	0.0684 (7)	
H13	0.5834	0.6716	0.1048	0.082*	
C14	0.3827 (3)	0.5431 (3)	0.1335 (2)	0.0574 (6)	
H14	0.4558	0.4364	0.1438	0.069*	

### Atomic displacement parameters (Å^2^)

**Table:**

	*U*^11^	*U*^22^	*U*^33^	*U*^12^	*U*^13^	*U*^23^
S1	0.0624 (4)	0.0385 (3)	0.0468 (4)	−0.0171 (3)	−0.0074 (3)	0.0017 (2)
Cl1	0.0899 (5)	0.0429 (3)	0.1012 (6)	−0.0076 (3)	−0.0297 (4)	−0.0227 (3)
Cl2	0.0746 (4)	0.0417 (3)	0.0626 (4)	−0.0171 (3)	−0.0078 (3)	−0.0088 (3)
O1	0.1015 (13)	0.0397 (8)	0.0520 (10)	−0.0200 (8)	0.0019 (9)	−0.0110 (7)
O2	0.0585 (10)	0.0693 (10)	0.0709 (12)	−0.0269 (8)	−0.0199 (9)	0.0141 (8)
C1	0.0487 (13)	0.0397 (11)	0.0445 (14)	−0.0069 (10)	0.0002 (10)	−0.0040 (10)
C2	0.0411 (12)	0.0379 (11)	0.0488 (14)	−0.0107 (9)	0.0018 (10)	−0.0047 (10)
C3	0.0321 (11)	0.0369 (10)	0.0441 (13)	−0.0055 (8)	−0.0011 (9)	−0.0031 (9)
C4	0.0359 (12)	0.0397 (11)	0.0488 (14)	−0.0095 (9)	−0.0015 (10)	−0.0042 (10)
C5	0.0530 (14)	0.0442 (12)	0.0578 (16)	−0.0106 (10)	−0.0052 (11)	0.0051 (11)
C6	0.0648 (17)	0.0724 (17)	0.0435 (16)	−0.0043 (13)	−0.0111 (12)	0.0009 (12)
C7	0.0566 (16)	0.0715 (16)	0.0539 (17)	−0.0025 (13)	−0.0132 (12)	−0.0226 (13)
C8	0.0426 (13)	0.0448 (12)	0.0593 (16)	−0.0020 (10)	−0.0073 (11)	−0.0147 (11)
C9	0.0524 (14)	0.0362 (11)	0.0372 (13)	−0.0095 (10)	−0.0051 (10)	−0.0043 (9)
C10	0.0523 (14)	0.0432 (12)	0.0685 (17)	−0.0047 (11)	−0.0138 (12)	−0.0070 (11)
C11	0.091 (2)	0.0358 (12)	0.0729 (19)	−0.0110 (13)	−0.0263 (16)	−0.0023 (11)
C12	0.096 (2)	0.0589 (16)	0.0488 (16)	−0.0414 (15)	−0.0048 (14)	−0.0056 (12)
C13	0.0584 (16)	0.0802 (18)	0.079 (2)	−0.0298 (14)	0.0101 (14)	−0.0331 (15)
C14	0.0552 (15)	0.0480 (12)	0.0706 (18)	−0.0073 (11)	−0.0029 (12)	−0.0187 (12)

### Geometric parameters (Å, °)

**Table:**

S1—O2	1.4353 (16)	C6—C7	1.380 (3)
S1—O1	1.4364 (15)	C6—H6	0.9300
S1—C1	1.748 (2)	C7—C8	1.373 (3)
S1—C9	1.7683 (19)	C7—H7	0.9300
Cl1—C8	1.738 (2)	C9—C10	1.369 (3)
Cl2—C4	1.736 (2)	C9—C14	1.373 (3)
C1—C2	1.320 (3)	C10—C11	1.381 (3)
C1—H1	0.9300	C10—H10	0.9300
C2—C3	1.464 (3)	C11—C12	1.360 (3)
C2—H2	0.9300	C11—H11	0.9300
C3—C8	1.398 (3)	C12—C13	1.367 (3)
C3—C4	1.404 (3)	C12—H12	0.9300
C4—C5	1.365 (3)	C13—C14	1.378 (3)
C5—C6	1.371 (3)	C13—H13	0.9300
C5—H5	0.9300	C14—H14	0.9300
			
O2—S1—O1	119.35 (10)	C8—C7—C6	120.1 (2)
O2—S1—C1	107.76 (10)	C8—C7—H7	119.9
O1—S1—C1	108.27 (9)	C6—C7—H7	119.9
O2—S1—C9	108.45 (9)	C7—C8—C3	122.2 (2)
O1—S1—C9	108.92 (9)	C7—C8—Cl1	117.54 (16)
C1—S1—C9	102.84 (9)	C3—C8—Cl1	120.25 (17)
C2—C1—S1	121.25 (17)	C10—C9—C14	120.77 (19)
C2—C1—H1	119.4	C10—C9—S1	119.66 (16)
S1—C1—H1	119.4	C14—C9—S1	119.57 (15)
C1—C2—C3	127.63 (19)	C9—C10—C11	119.2 (2)
C1—C2—H2	116.2	C9—C10—H10	120.4
C3—C2—H2	116.2	C11—C10—H10	120.4
C8—C3—C4	115.12 (19)	C12—C11—C10	120.2 (2)
C8—C3—C2	125.15 (17)	C12—C11—H11	119.9
C4—C3—C2	119.72 (17)	C10—C11—H11	119.9
C5—C4—C3	123.29 (18)	C11—C12—C13	120.5 (2)
C5—C4—Cl2	118.16 (15)	C11—C12—H12	119.7
C3—C4—Cl2	118.52 (16)	C13—C12—H12	119.7
C4—C5—C6	119.5 (2)	C12—C13—C14	120.0 (2)
C4—C5—H5	120.3	C12—C13—H13	120.0
C6—C5—H5	120.3	C14—C13—H13	120.0
C5—C6—C7	119.8 (2)	C9—C14—C13	119.3 (2)
C5—C6—H6	120.1	C9—C14—H14	120.3
C7—C6—H6	120.1	C13—C14—H14	120.3
			
O2—S1—C1—C2	128.08 (17)	C2—C3—C8—C7	−179.5 (2)
O1—S1—C1—C2	−2.3 (2)	C4—C3—C8—Cl1	177.68 (14)
C9—S1—C1—C2	−117.47 (18)	C2—C3—C8—Cl1	−1.1 (3)
S1—C1—C2—C3	−177.57 (14)	O2—S1—C9—C10	10.8 (2)
C1—C2—C3—C8	−40.8 (3)	O1—S1—C9—C10	142.12 (18)
C1—C2—C3—C4	140.4 (2)	C1—S1—C9—C10	−103.16 (19)
C8—C3—C4—C5	1.1 (3)	O2—S1—C9—C14	−169.41 (17)
C2—C3—C4—C5	179.93 (18)	O1—S1—C9—C14	−38.06 (19)
C8—C3—C4—Cl2	179.04 (14)	C1—S1—C9—C14	76.65 (18)
C2—C3—C4—Cl2	−2.1 (3)	C14—C9—C10—C11	−0.9 (3)
C3—C4—C5—C6	−0.3 (3)	S1—C9—C10—C11	178.95 (17)
Cl2—C4—C5—C6	−178.28 (17)	C9—C10—C11—C12	1.0 (3)
C4—C5—C6—C7	−0.8 (3)	C10—C11—C12—C13	0.0 (4)
C5—C6—C7—C8	1.1 (4)	C11—C12—C13—C14	−1.1 (4)
C6—C7—C8—C3	−0.3 (3)	C10—C9—C14—C13	−0.2 (3)
C6—C7—C8—Cl1	−178.79 (18)	S1—C9—C14—C13	179.95 (18)
C4—C3—C8—C7	−0.8 (3)	C12—C13—C14—C9	1.2 (4)
